# Resveratrol attenuates TLR-4 mediated inflammation and elicits therapeutic potential in models of sepsis

**DOI:** 10.1038/s41598-020-74578-9

**Published:** 2020-11-02

**Authors:** Binbin Wang, Gregory Lucien Bellot, Kartini Iskandar, Tsung Wen Chong, Fera Yiqian Goh, June Jingyi Tai, Herbert Schwarz, Siew Cheng Wong, Shazib Pervaiz

**Affiliations:** 1 Department of Physiology, Yong Loo Lin School of Medicine, National University of Singapore, Singapore, 117597 Singapore; 2 NUS Graduate School for Integrative Sciences and Engineering, National University of Singapore, Singapore, Singapore; 3 Department of Hand & Reconstructive Microsurgery, University Orthopedic, Hand & Reconstructive Microsurgery Cluster, National University Health System, Singapore, Singapore; 4 Department of Urology, Singapore General Hospital (SGH), Singapore, Singapore; 5 Singapore Immunology Network (SIgN), Agency for Science, Technology and Research (ASTAR), Singapore, 138648 Singapore; 6 Department of Biochemistry, National University of Singapore, Singapore, Singapore; 7 National University Cancer Institute, National University Health System, Singapore, Singapore; 8 Faculte de Medicine, University of Paris, Paris, France

**Keywords:** Autophagy, Inflammatory diseases

## Abstract

Sepsis is a potentially fatal condition triggered by systemic inflammatory response to infection. Due to the heightened immune reactivity and multi-organ pathology, treatment options are limited and several clinical trials have not produced the desired outcome, hence the interest in the discovery of novel therapeutic strategies. The polyphenol resveratrol (RSV) has shown promise against several pathological states, including acute and chronic inflammation. In this study, we evaluated its therapeutic potential in a murine model of sepsis and in patients undergoing transrectal ultrasound biopsy. RSV was able to inhibit lipopolysaccharide (LPS) stimulated inflammatory responses through blocking Phospholipase D (PLD) and its downstream signaling molecules SphK1, ERK1/2 and NF-κB. In addition, RSV treatment resulted in the downregulation of MyD88, an adaptor molecule in the TLR4 signaling pathway, and this effect at least in part, involved RSV-induced autophagy. Notably, RSV protected mice against polymicrobial septic shock induced upon cecal ligation and puncture, and inhibited pro-inflammatory cytokine production by human monocytes from transrectal ultrasound (TRUS) biopsy patients. Together, these findings demonstrate the immune regulatory activity of RSV and highlight its therapeutic potential in the management of sepsis.

## Introduction

Despite scientific investigations and clinical studies sepsis remains a poorly understood medical condition with limited therapeutic options. Sepsis is triggered by a response to an infection and characterized by an initial heightened immune response that can potentially lead to systemic damages to organs with potential life threatening consequences. The high incidence of severe sepsis reaches around 20 million cases yearly with approximately a 40% mortality rate despite the researches that have been focusing on this condition and thus remains a medical concern of high priority^1^. The early acute inflammatory response is classically instigated by neutrophils, monocytes and macrophages that express receptors on their surface named PRRs (Pattern Recognition Receptors). These PRRs can bind evolutionary-conserved molecular motifs called PAMPs (pathogen-associated molecular patterns) displayed by microorganisms. The binding of LPS, a common PAMP present in Gram-negative bacteria, to TLR4 is believed to be an important event involved in sepsis’ inflammatory response and thus represents an essential signaling pathway to investigate in order to potentially establish new therapeutic possibilities^2,3^. In addition to TLRs stimulation, other inflammatory mediators have been identified in sepsis such as C5a, MIF, HMGB1 and IL17-A^1,4^. Heightened production of proinflammatory factors such as IL-6, IL-1β and TNFα may trigger an over stimulation of the immune system which can in turn lead to severe tissue damage. In addition, chemokines such as MCP1 and MIP-1α display chemo-attractive properties for the immune cells. HMGB1, initially described as a transcription factor, is also a critical mediator involved in sepsis and a sustained HMGB1 level has been correlated with a higher mortality rate^5^. Furthermore, several studies also showed key signaling mechanisms involving PLD and Sphingosine Kinase (SphK) activities in immune cells as well as in in vivo inflammatory disease models^6–11^. Lee et al. suggested a major role for PLD2 in neutrophils in the onset of sepsis in experimental models^8^.

Resveratrol (*trans*-3,5,4′-trihydroxystilbene) is an active component of stilbene phytoalexins^12–14^. It is a polyphenolic and non-flavonoid molecule displaying potent antioxidant properties present in the skin of fruits such as grapes and berries. Besides its antioxidant capabilities, studies also showed that RSV exhibits anti-proliferative and anti-inflammatory effects in several disease models^15–17^. Investigations showed that RSV diminishes edema, airway inflammation, endotoxemia-induced acute phase response and osteoarthritis. Numerous investigations on molecular mechanisms aimed to decipher the anti-inflammatory properties of RSV. One such mechanism involves the inhibition of cyclooxygenase (COX) activity, either through regulating COX transcription or through binding directly to COX-2 protein^18,19^. RSV can also modulate NF-κB signaling by blocking IκB phosphorylation that promotes NF-κB translocation to the nucleus^20,21^. There is also experimental evidence that RSV downregulates PLD activity and degranulation by stimulating human neutrophils^6^. Furthermore, Bereswill et al. reported that oral treatment with RSV can alleviate Th1-dependent immune response, thereby reducing acute inflammation in the small intestine^22^.

As many of the pathways activated during the development of sepsis contain signaling nodes that have been shown in other model systems to be regulated by RSV, we attempted here to study the therapeutic potential of RSV using in vitro and in vivo models. While studying the effect on immune mediators, we also investigated the role of autophagy in immune-modulation by RSV. To that end, RSV is a potent inducer of autophagy^23,24^, and a number of mediators have been implicated in this signaling^25–27^. Pertinently, stimuli that induce autophagy have shown promise in models of sepsis, which could be attributed to the protective effect of autophagy via mechanisms that prevent immunosuppression associated with sepsis^28,29^. Autophagy is initiated early after the onset of sepsis and mainly involves the AMPK and MAPK signaling pathways upon TLR4 and TLR9 activation^30–32^. We therefore postulated that RSV-induced autophagy may play a role that intersects with TLR4 signaling components to regulate systemic inflammation associated with sepsis. Here we provide evidence that RSV not only down-modulates TLR4-activated SphK, NF-κB and ERK1/2 by inhibiting PLD activity, but also downregulates MyD88, which could be rescued, at least in part, upon inhibition of autophagy. More importantly, these findings highlight the therapeutic potential of RSV in a murine model of sepsis and in monocytes derived from patients undergoing TRUS biopsies.

## Materials and methods

### Blood sample collection

The blood collection protocol for this study was approved by the SingHealth Centralised Institutional Review Board of Singapore and performed according its guidelines. Patients undergoing transrectal ultrasound (TRUS) biopsy were recruited and blood samples were harvested before the biopsy procedure and 24 h post biopsy (CIRB 2009/502/D). Human peripheral blood monocytes were obtained from healthy donors at the National University Hospital Blood Donation Centre (National University Hospital, Singapore) according to a protocol approved by the Institutional review Board of the National University of Singapore (protocol 07-005E). Informed consents from patients and volunteers were obtained for this study according to the approved protocols.

### Measurement of Cytokines, chemokines and HMGB1

2 × 10^5^ monocytes per sample were pretreated for 2 h with RSV (10–40 μM) and then stimulated for 24 h with 100 ng/ml LPS from *Salmonella typhimurim* (Calbiochem, Merck KGaA, Darmstadt, Germany) with supernatant collection performed at different time points before being stored at -20 °C. For the TRUS patients, 3 × 10^5^ monocytes were plated per well and either left untreated or treated with 20 μM or 40 μM RSV for 24 h before collecting the supernatants. Measurement of IL-6, TNFα, IL-1β, and MCP-1 concentrations was performed with OptEIA™ Kit (BD Biosciences, San Jose, CA, USA). DuoSet ELISA Development System (R&D System, USA) was used to measure MIP-1α. HMGB1 levels were measured using Shino-Test’s HMGB1 ELISA kit II (Shino-Test Corporation, Japan). Cytokines and chemokines in the mouse model were measured using similar methods.

### Determination of SphK and PLD enzymatic activities

The protocol for SphK activity was adapted from Billich et al.^10,33^ . Briefly, cells were suspended in ice-cold extraction buffer (0.1 M phosphate buffer pH 7.4, 20% glycerol, 1 mM 2β-mercaptoethanol, 1 mM EDTA, 20 mM ZnCl_2_, 1 mM sodium orthovanadate, and 15 mM sodium fluoride and protease inhibitors cocktail) before being submitted to freeze and thaw cycles. The protein extracts were then incubated with a reaction buffer (20 μM of 15-NBD-Sph prepared as a complex with BSA, 1 mM ATP and 50 mM 4-(2-hydroxyethyl)piperazine-1-ethanesulfonic acid, pH 7.4, containing 15 mM MgCl_2_, 0.005% Triton X-100, 10 mM KCl) for 30 min at 37 °C. Quenching of the reaction was done using 1 M potassium phosphate buffer and chloroform/methanol at 2:1 ratio. Phases were separated by centrifugation. A fraction from the aqueous layer was transferred into a white 96-well polystyrene microplate and dimethylformamide was added to the mix. Fluorescence within the samples was then measured at 485/535 nm using a spectrophotometer (TECAN Spectrophor Plus). A reaction mixture without extract served as a blank.

PLD activity was measured through a standard transphosphatidylation assay^10,34^. Primary monocytes (10^6^ cells/ml) were incubated with [^3^H] palmitic acid (5 µCi/ml; Amersham Biosciences, UK) in culture medium for 16 h for the radiolabeling step. Cells were washed and incubated for another 15 min in medium supplemented with 0.3% ethanol before LPS stimulation for 30 min. After cell lysis a Bligh-Dyer phase separation method was performed for lipids extraction before measuring phosphatidylethanol accumulation as described previously^34^.

### Poly acrylamide gel electrophoresis (PAGE) and Western blot analysis

Whole cell lysates (40 μg) were loaded and resolved using 12% acrylamide SDS-PAGE and transferred to 0.45 μm polyvinylidene difluoride (PVDF) membranes (Merck Millipore, Darmstadt, Germany). The membranes were blotted using the following primary antibodies: rabbit anti-LC3B, anti-phospho-ERK1/2, anti-total-ERK1/2, anti-PARP, mouse anti-MyD88 (Cell Signaling Technology, Danvers, MA, USA) and anti-p62/SQSTM1 (Santa Cruz Biotechnology, Dallas, TX, USA). Detection of the proteins of interest was achieved by incubating the membranes with secondary antibodies (anti-IgG HRP-conjugated) and using a chemiluminescence kit (ECL detection system, Amersham Biosciences, UK).

### Determination of NF-κB DNA-binding activity

Cells were pre-treated for 15 min with 40 µM RSV followed by stimulation with LPS (100 ng/ml) for 30 min, using the EZ-Detect transcription factor kit (Thermo Scientific Pierce, Rockford, IL). NF-κB activity was measured with 20 µg of nuclear extracts as instructed by the kit’s protocol. All samples were run in triplicate.

### Cell migration assay

Cell migration was assayed using the Chemicon QCMTM Chemotaxis 3 µm 96-well Cell Migration Assay kit (Chemicon, Temecula, CA, USA). The upper chamber was seeded with the tested cells and medium containing 100 ng/ml LPS was used in the lower chamber before incubation at 37 °C for 2-4 h. Cells present in the lower chamber were harvested while the cells attached to the interface membrane were dissociated with Cell Detachment Buffer (0.05% trypsin in Hanks Balanced Salt Solution (HBSS) containing 25 mM HEPES) to be collected as well. Cells collected were lysed and their number was determined through the use of CyQuant GR dye (Molecular Probes, Eugene, OR, USA). The dye emits fluorescence when binding to nucleic acids and when compared to a standard curve allows to determine the cell number based on the fluorescence measured (Ex 480 nm/Em 520 nm).

### Cecal ligation and puncture (CLP) induced sepsis in mice

Male C57/BL6 mice aged from 8 to 10 weeks (Laboratory Animal Centre, Sembawang, Singapore) were used as a model for this study. Experimental procedures followed the NUS guidelines for animal experimentation and were approved by the Institutional Animal Care and Use Committee (IACUC, National University of Singapore). Sepsis was induced by CLP as previously described^35^. Following anesthesia using ketamine (75 mg/kg, intramuscular injection) and xylazine (20 mg/kg), a midline abdominal surgical incision was made . Cecum was then isolated and ligated at 5 mm from the cecal tip, away from the ileocecal valve, and the ligated cecal stump was then pierced by two “through and through” punctures (22-gauge needles). After repositioning the cecum into the intra-abdominal space, the abdomen was closed with a running suture of 5–0 prolene. Similar operating procedure was performed on sham mice without the CLP step. 1 mg/kg body weight of RSV (diluted in 200 μl sterile PBS) was injected 1 h, 3 h and 6 h after CLP procedure in the RSV-treated group. 200 μl sterile PBS was injected at the corresponding time points in the control group. 24 h after CLP all animals were sacrificed and blood, peritoneal lavage and organs were harvested for analysis.

### Determination of bacterial counts

10 µl of collected blood and peritoneal lavage, non-diluted or diluted in PBS, were spread onto Petri dishes (Trypticase Soy Agar with 5% Sheep Blood) and cultured at 37 °C for 24 h before counting the colony forming units (CFU).

### Measurement of peritoneal leukocytes infiltration

Leukocytes obtained from the different groups of animals were pooled, washed and adjusted to a concentration of 6 × 10^6^cells/ml. After adding 0.1 ml of the leukocyte preparation to 0.1 ml of FCS, a cytocentrifugation was performed (450 rpm, 3 min). Cells were then transferred to slides and stained with Giemsa. Two independent observers performed a differential cell counts from randomly selected fields.

### Fluorescent confocal microscopy

Autophagosomes formation, LC3 and MyD88 localization were evaluated using confocal microscopy. Following RSV treatment, U-937 cells were fixed with 4% paraformaldehyde and mounted on microscope slides using a cytospin centrifuge. The subsequent permeabilization was performed by treating the cells with 0.1% Triton X-100 in PBS for 2 min followed by a blocking step with 1% BSA (1 h at room temperature). Incubation with primary antibodies rabbit anti-LC3B and mouse anti-MyD88 (Cell Signaling Technology, Danvers, MA, USA) was done at room temperature for 2 h and was followed by a washing step with PBS preceding the final incubation with Alexa-fluoro-conjugated IgG based secondary antibodies. After three washes with PBS coverslips were mounted on glass slides and imaging was done using an Olympus Fluoview FV1000 confocal microscope. Analysis of images was achieved using Olympus Fluoview 2.0 viewer.

### Transmission electron microscopy

Treated or untreated U-937 cells were fixed with 2.5% glutaraldehyde (4 °C, overnight). After washing with PBS three times, cells were incubated with 1% osmium tetroxide for 1 h on a rotator for the post-fixation step. After three washing steps cells were resuspended in 6% gelatin for 10 min and pelleted at 3000 rpm. Excess of gelatin was removed and the cells were submitted to a new fixation step in 2.5% glutaraldehyde for another 10 min. The samples were washed three times with distilled water before being cut into 1mm^3^ cubes for further processing. Cubes dehydration was achieved using solutions of increasing ethanol concentrations (from 25 to 100% alcohol and then 100% acetone) before being progressively permeated with Epon-Araldite (from 1:6 ratio of Epon-Araldite: acetone to 100% Epon-Araldite). Three Epon-Araldite baths were made on the following day (40–50 °C). Polymerization was performed (60 °C for 24 h). Sample’s Sects. (90-100 nm) were prepared and collected on slot copper grids using a Leica EM UC6 microtome. Counter-staining was performed using uranyl acetate and lead citrate and then observed using a Philips transmission electron microscope JEOL JEM-1010 (100 keV electron beam energy). Pictures acquisition was done with a Gatan ES500W Erlangshen camera (1350 × 1040 pixels).

### Statistical analysis

Data are presented as mean ± SD where n ≥ 3. Statistical significance was determined using one-way ANOVA, two-way ANOVA and two-tailed unpaired/paired student’s *t*-test as indicated in the figures legends with *p* value of less than 0.05 considered statistically significant.

### Ethics approval and consent to participate

Consent and recruitment of patients undergoing transrectal ultrasound (TRUS) biopsy was performed according to the study’s protocol approved by the Institutional Review Board, Singapore in accordance to the guidelines of the SingHealth Centralised institutional review board of Singapore (ref. number CIRB 2009/502/D). Human peripheral blood monocytes were obtained with consent from anonymous healthy volunteer donors at the National University Hospital Blood Donation Centre (National University Hospital, Singapore) according to the study’s protocol approved by the National University of Singapore Institutional Review Board (Ref. number 07-005E).

The animal study was conducted according to the NUS guidelines for animal experimentation and project licenses approved by the National University of Singapore.

## Results

### RSV inhibits LPS-mediated cytokine and chemokine production, HMGB1 release and leukocyte migration

**Figure 1:**
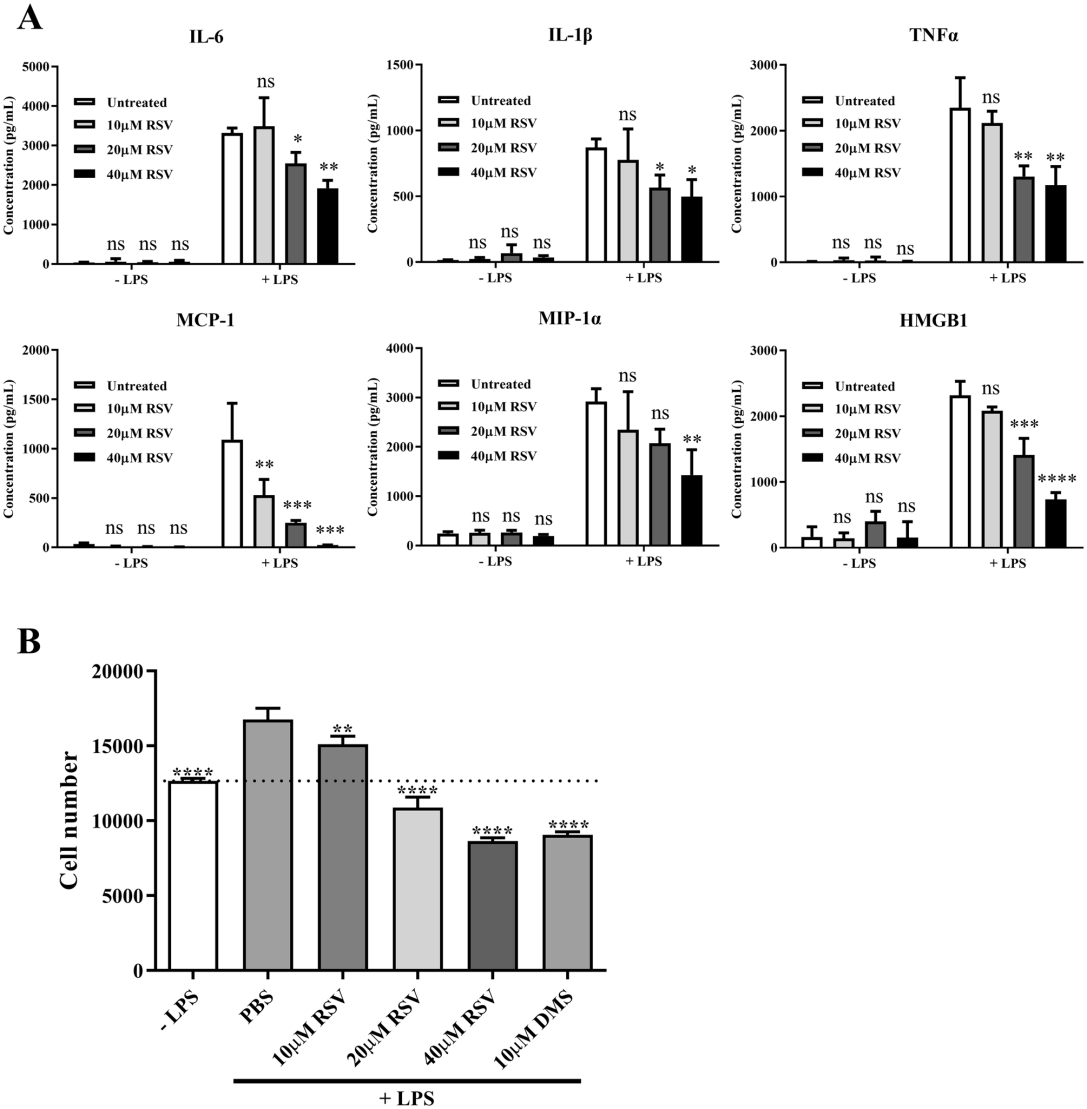
RSV inhibits LPS-triggered functional responses such as pro-inflammatory cytokine, chemokine and HMGB1 production as well as monocyte migration in primary monocytes. **(A**) IL-6, IL-1β, TNFα, MCP-1, MIP-1α and HMGB1 levels using ELISA in human primary monocytes, untreated or pre-treated with indicated doses of RSV, with or without LPS stimulation (100 ng/ml) for 24 h (*p < 0.05, **p < 0.01, ***p < 0.001, ****p < 0.001). n = 3 for each condition and statistical analysis was performed using two-way ANOVA followed by post hoc Dunnett’s multiple comparisons test. (**B**) Primary monocytes were left untreated or pre-treated with varying doses of RSV or 10 μM DMS (n = 3 for each condition) and statistical analysis was performed using one-way ANOVA followed by post hoc Dunnett’s multiple comparisons test. The number of monocytes migrated in the presence of LPS without RSV treatment were compared with control samples in the absence of LPS. The numbers of monocytes migrated in the RSV or the sphingosine kinase inhibitor N,N-Dimethylsphingosine (DMS) pre-treated wells towards LPS were compared with PBS-treated samples towards LPS (LPS + PBS) (**p < 0.01, ****p < 0.0001). Data were derived from Dr. Wang Binbin PhD Thesis ^36^.

The initial inflammatory phase in sepsis is usually characterized by systemic inflammation and organ failure. Here we started by investigating the effect of RSV on cytokine and chemokine production in human primary monocytes. The results showed that RSV was able to inhibit LPS-triggered TNFα, IL-1β, IL-6, MCP-1, MIP1α and HMGB1 production in a dose-dependent manner (Fig. 1A). In the presence of 40 µM RSV, the production of TNFα, IL-1β, MIP-1α and IL-6 by LPS-stimulated cells was reduced more than 40% of the LPS only treated samples. Strikingly, a much stronger effect was observed on MCP-1 and HMGB1 with virtually a complete inhibition at the highest RSV concentration compared to LPS alone (Fig. 1A). In addition, pre-incubation of primary monocytes with 10–40 µM of RSV inhibited LPS-induced monocyte migration in a dose-dependent manner (Fig. 1B)^36^. These results indicate that RSV attenuates LPS-stimulated cytokine, chemokine and HMGB1 production as well as migration in human primary blood monocytes.

### RSV blocks LPS-induced NF-κB activation, SphK activity and ERK1/2 phosphorylation

**Figure 2:**
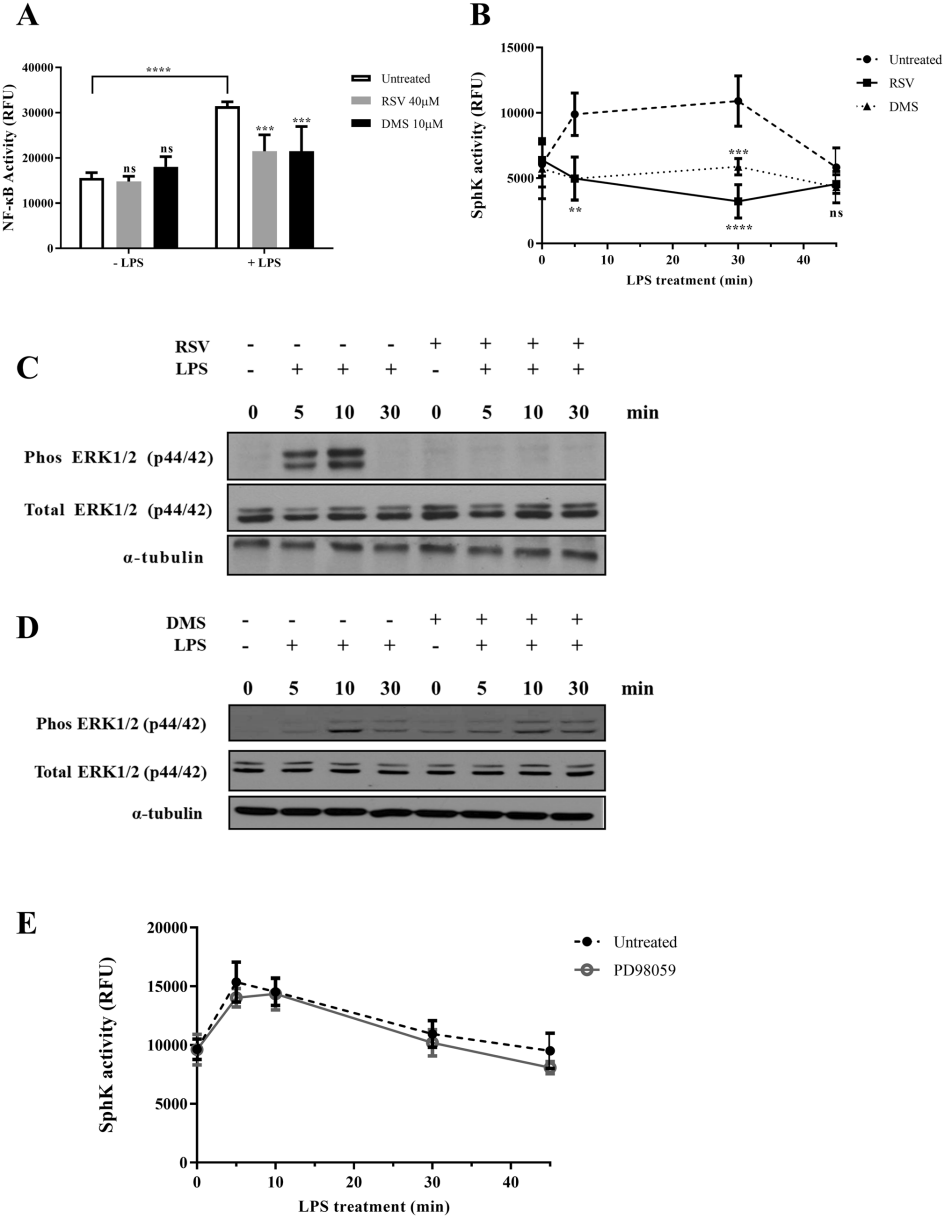
RSV blocks LPS-induced NFκB activation, SphK activity and ERK1/2 phosphorylation. **(A**) NF-κB activity in untreated cells or following LPS (100 ng/ml) stimulation for 30 min (LPS) was assessed using the NF-κB kit (***p < 0.001, ****p < 0.0001); n = 5 for each condition and two-way ANOVA followed by post hoc Sidak’s multiple comparisons test. (**B**) SphK activity of cells without LPS stimulation or stimulated with 100 ng/ml LPS for 5, 10, 30 and 45 min in PBS, RSV or DMS pre-treated groups (**p < 0.01, ***p < 0.001, ****p < 0.0001); n = 3 for each condition and statistical analysis was performed using two-way ANOVA followed by post hoc Dunnett’s multiple comparisons test. (**C**) Immunoblot analysis of expression of phosphorylated ERK1/2 in human primary monocytes treated with or without 40 µM RSV and then stimulated with 100 ng/ml LPS for 5, 10 and 30 min, total ERK1/2 and α-tubulin serve as loading controls. (**D**) Immunoblot analysis of expression of phosphorylated ERK1/2 in human primary monocytes treated with or without 10 µM DMS and then triggered with 100 ng/ml LPS for 5, 10 and 30 min, total ERK1/2 and α-tubulin serve as loading controls. Note: western blots were performed on the same membranes cut in different strips based on molecular weight to probe for the desired targets. (**E**) SphK activity of cells without LPS stimulation or stimulated with 100 ng/ml LPS for 5, 10, 30 and 45 min in both untreated group or PD98059 pre-treated group (n = 3 for each condition). Statistical comparison performed for each time point between untreated and treated conditions. Data were derived from Dr. Wang Binbin PhD Thesis ^36^.

The expression of pro-inflammatory cytokines and chemokines induced by LPS is regulated through the TLR-4 signaling pathways via the transcription factor, NF-κB^37,38^. We questioned if RSV-induced inhibition of pro-inflammatory mediators was a function of its ability to regulate NF-κB activity. Indeed, a priori treatment of primary monocytes with 40 µM RSV for 15 min inhibited LPS-induced NF-κB activation (Fig. 2A), as assessed by an ELISA assay system described previously^39^. Earlier studies also reported the involvement of SphK in signaling pathways activated by pro-inflammatory signals such as LPS and C5a^40,41^. This was confirmed using N,N-Dimethylsphingosine (DMS), a SphK inhibitor. Pre-treatment with 10 µM DMS effectively blocked LPS-stimulated monocyte migration (Fig. 1B) as well as NF-κB activity (Fig. 2A)^36^. Prompted by these data, we hypothesized that the inhibition of NF-κB activity by RSV could be modulated through its effect on SphK activity. Of note, the rapid increase in SphK activity in LPS-treated monocytes was abolished in cells pre-treated with 40 µM RSV (Fig. 2B)^36^.

Aside from the lipid mediators, activation of Extracellular signal-Regulated Kinases (ERKs) has also been documented in neutrophils, monocytes and macrophages in response to inflammatory stimuli. In this regard, ERK1/2 activation is induced by C5a in neutrophils, and by TNFα or LPS in primary monocytes^10,42^. Consistent with the latter, ERK1/2 phosphorylation was induced upon exposure of monocytes to LPS, which was completely abolished upon pre-treatment with 40 µM RSV for 15 min (Fig. 2C), but not upon incubation with 10 µM DMS (Fig. 2D)^36^. Reciprocally, pharmacological inhibition of ERK1/2 (PD98059; 20 µM) did not affect LPS-induced SphK activity, thereby indicating that the stimulatory effect of LPS on SphK activity is independent of ERK1/2 (Fig. 2E)^36^.

### PLD is implicated in the regulation of LPS-induced SphK activity by RSV

**Figure 3:**
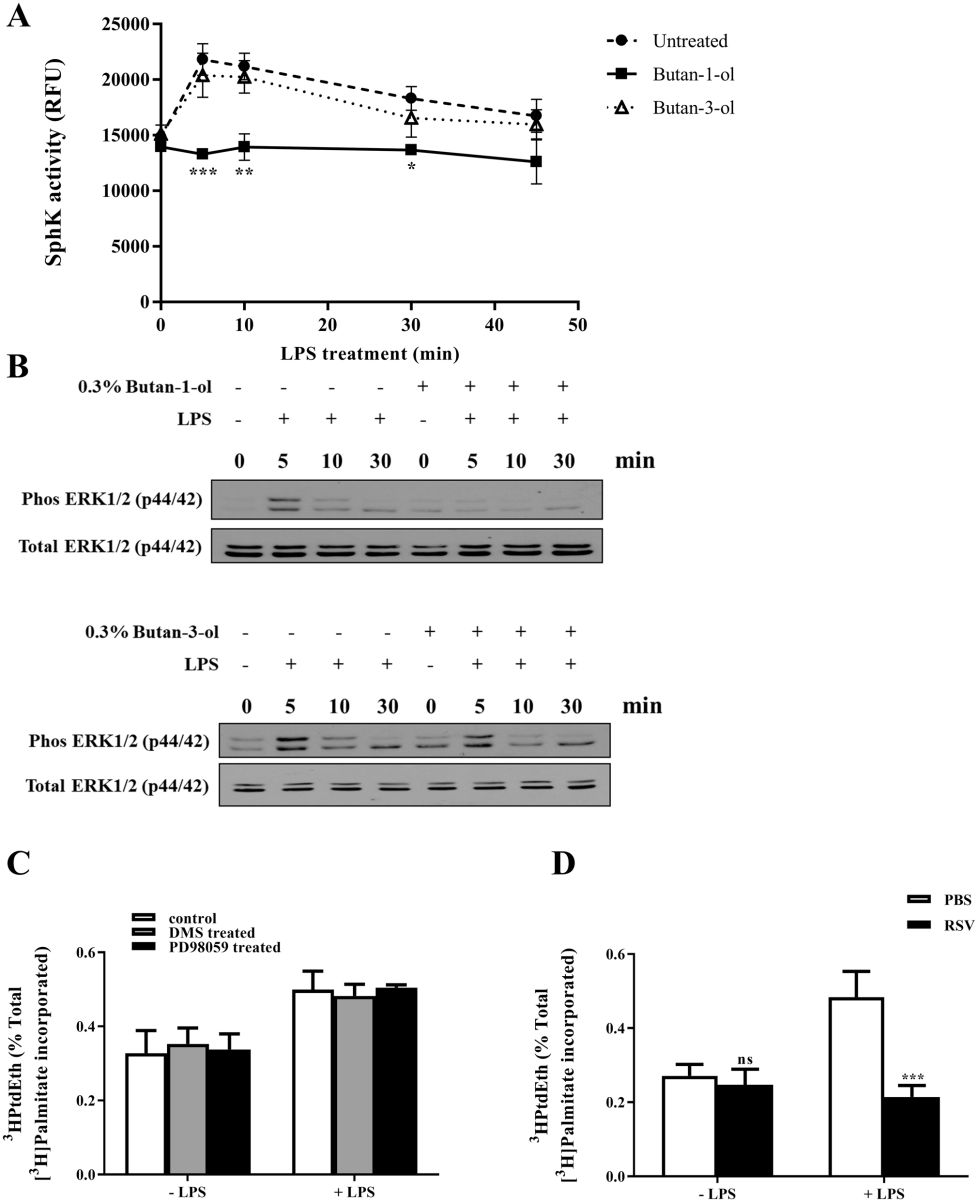
Both LPS-triggered SphK activity and ERK1/2 phosphorylation are downstream of PLD activity which can be blocked by RSV. **(A**) SphK activity of cells with or without LPS stimulation 100 ng/ml LPS for 5, 10, 30 and 45 min in PBS group, butan-1-ol pretreatment group or butan-3-ol pretreatment group (n = 2 for each condition; *p < 0.05, **p < 0.01, ***p < 0.001). Statistical analysis was performed using two-way ANOVA followed by post hoc Dunnett’s multiple comparisons test. (**B**) Immunoblot analysis of expression of phosphorylated ERK1/2 in human primary monocytes treated with or without 0.3% butan-1-ol or 0.3% butan-3-ol before stimulation with 100 ng/ml LPS for 5, 10 and 30 min, total ERK1/2 serves as a loading control. (**C**) PLD activity of human monocytes measured following stimulation with LPS (100 ng/ml) for 30 min with pre-treatment with PBS, 10 µM DMS or 20 µM PD98059 for 15 min. Samples stimulated with LPS are compared with the samples without LPS stimulation for each treatment group (n = 3 for each condition). (**D**) PLD activity of human monocytes was measured following LPS treatment (100 ng/ml) for 30 min with pre-treatment with PBS or 40 µM RSV (n = 3 for each condition; ***p < 0.001). Statistical analysis was performed using two-way ANOVA followed by post hoc Sidak’s multiple comparisons test. Note: western blots were performed on the same membranes cut in different strips based on molecular weight to probe for the desired targets. Data were derived from Dr. Wang Binbin PhD Thesis ^36^.

It was previously reported that PLD can regulate SphK activity and ERK1/2 phosphorylation upon TNFα receptor activation ^40,43^. Given our observations, we first set out to determine if LPS-dependent SphK activity and ERK1/2 phosphorylation in our model system were also regulated by PLD. Results showed that pre-incubation of human primary monocytes with the PLD inhibitor, butan-1-ol (0.3%), significantly blocked SphK activity and ERK1/2 phosphorylation upon LPS stimulation (Fig. 3A,B)^36^. Moreover, the inability of DMS or PD98059 to block LPS-induced PLD activity argues in favor of an upstream role of PLD in regulating SphK and ERK activation upon LPS exposure (Fig. 3C)^36^. Importantly, pre-treatment of monocytes with 40 µM RSV inhibited LPS-induced PLD activity (Fig. 3D)^36^.

### RSV treatment protects mice against CLP-induced sepsis

**Figure 4:**
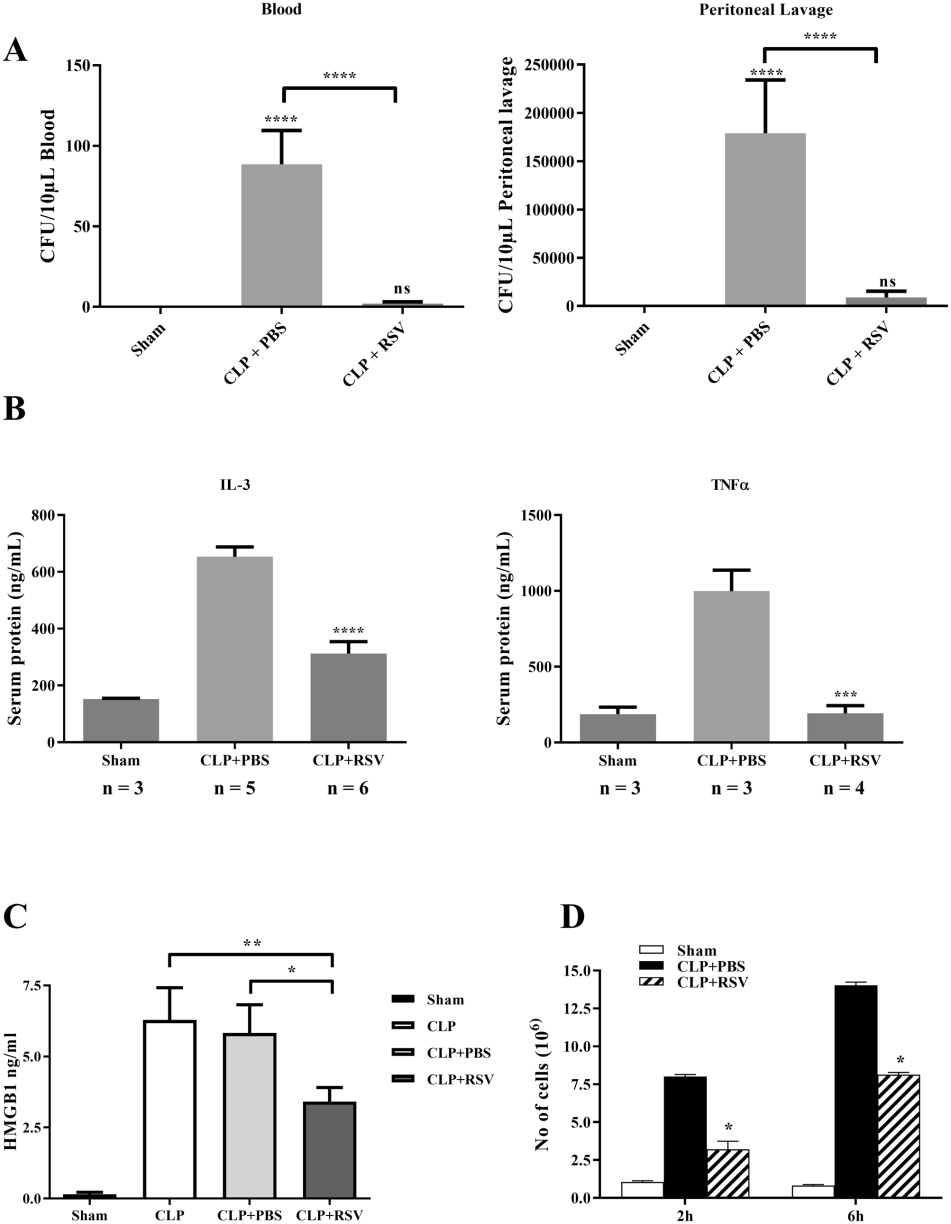
RSV is protective against CLP model of polymicrobial sepsis. **(A**) Bacteria counts in whole blood and peritoneal lavage fluid of sham-operated mice, CLP mice injected with PBS (CLP + PBS) and CLP mice and injected with 1 mg/kg of RSV (CLP + RSV) were assessed by colony-forming unit method 24 h after CLP (n = 5 for each condition). Statistical analysis was performed using one-way ANOVA followed by post hoc Tukey’s multiple comparisons test. .PBS-treated mice were compared to RSV-treated mice (**** p < 0.0001). (**B**) IL-3 and TNFα in serum of mice treated as in A, were measured 24 h after CLP by ELISA (***p < 0.001, ****p < 0.0001). Statistical analysis was performed using one-way ANOVA followed by post hoc Tukey’s multiple comparisons test**. (C**) Serum HMGB1 levels of mice treated as in A were measure by ELISA 24 h after CLP (n = 3 for each condition; *p < 0.05; **p < 0.01). Statistical analysis was performed using one-way ANOVA followed by post hoc Tukey’s multiple comparisons test. (**D**) Numbers of neutrophils infiltrated into peritoneum of mice treated as in A were counted at 2 h and 6 h after CLP (n = 3 for each condition). Statistical analysis was performed using two-way ANOVA followed by post hoc Tukey’s multiple comparisons test RSV-treated group was compared to PBS-treated group (*p < 0.05). Data were derived from Dr. Wang Binbin PhD Thesis^36^.

So far our results have demonstrated that RSV inhibited LPS-induced SphK and ERK1/2 activities via PLD, resulting in diminished cytokine production in human monocytes. To evaluate the translational potential of these in vitro findings, we next made use of the CLP-induced murine model of peritonitis and systemic bacteremia, which closely mimics the human clinical septic syndrome^44,45^. To evaluate the therapeutic efficacy of RSV, mice were injected (intra-peritoneally) with 1 mg/kg body weight of RSV (diluted in 200 μl sterile PBS), 1 h, 3 h and 6 h following the induction of CLP. First, we evaluated the bacterial load in both blood and peritoneal lavage. Bacteria load were significantly reduced in both the blood and peritoneal lavage of RSV-treated CLP mice (Fig. 4A), thus indicating the ability of RSV to reduce local and systemic bacterial dissemination (systemic septicemia)^36^. In addition, the levels of pro-inflammatory cytokines, IL-3 and TNFα, were significantly lower in the serum of RSV-treated mice, compared to PBS-treated animals (Fig. 4B). The levels of IL-1β, IL-5, IL-6, MCP-1, IFNγ, MIP-1α, MIP-1β and CXCL1 (KC) did not change significantly (Suppl. Fig. S1). Additionally, HMGB1 reported to be a potent stimulator of pro-inflammatory cytokine synthesis in human monocytes and a key player in the second wave of inflammatory responses^5,46^, was significantly decreased in the serum of RSV-treated mice, compared to PBS-treated mice (Fig. 4C)^36^. These results are in agreement with the in vitro data presented in Fig. 1A.

In the CLP-induced sepsis model, neutrophils and monocytes are the first cell types to be recruited into the peritoneal cavity and are critical actors in the host defense^35^. However, massive infiltration of immune cells is detrimental to tissues and organs integrity. Therefore, we measured the neutrophils count recruited into the peritoneal lavage at 2 h and 6 h post-CLP. Results showed that RSV-treated mice had a significantly lower quantity of infiltrated neutrophils in the peritoneum than PBS-treated mice (Fig. 4D)^36^. Collectively, these results indicate that RSV is effective in vivo in mitigating the inflammatory response in a CLP-induced sepsis model.

### RSV reduced inflammatory cytokines production by human monocytes from patients undergoing prostate biopsies

**Figure 5:**
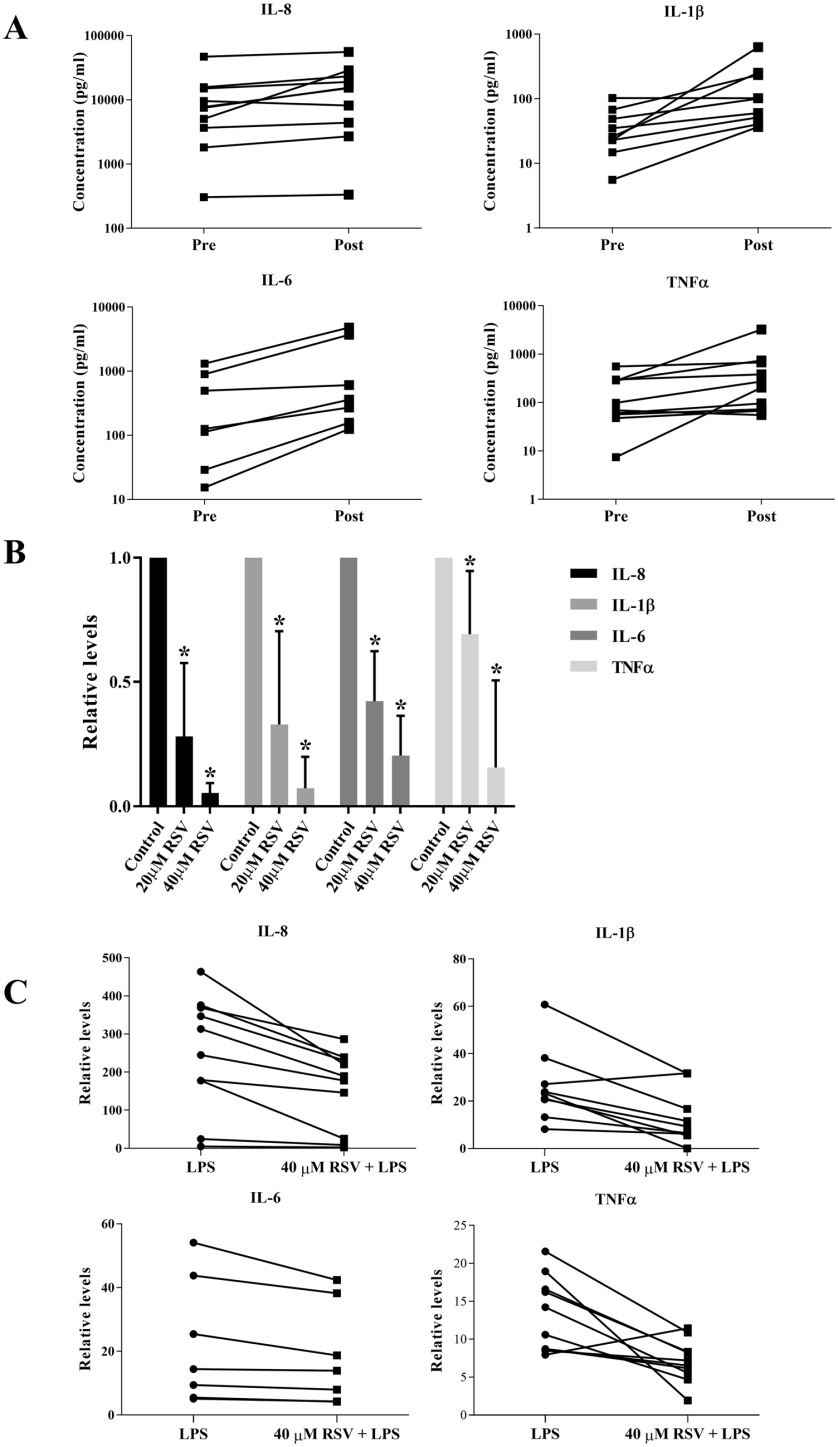
RSV inhibits pro-inflammatory cytokines production by human monocytes from post-TRUS biopsy patients. **(A)** Monocytes isolated from blood of patients before (Pre) and 24 h post-TRUS (Post) biopsies were cultured. Supernatants were harvested 24 h later to quantify for IL-8, TNF-α, IL-1β and IL-6 levels by ELISA. Each data point represents 1 patient sample and respective p-values shown are post-biopsy compared to pre-biopsy. Statistical analysis was performed using two-tailed paired t test. **(B**) Monocytes isolated from post-TRUS biopsy blood samples were either left untreated (Ctrl) or treated with 20 µM or 40 µM RSV. Supernatants were harvested 24 h later to quantify for IL-8, TNF-α, IL-1β and IL-6 levels by ELISA. Data shown are mean ± SD of n ≥ 4 and * depicts p < 0.05 compared to respective control samples. Statistical analysis was performed using one-way ANOVA followed by post hoc Tukey’s multiple comparisons test. **(C**) Monocytes from healthy donors either untreated or pre-treated with 40 µM RSV were stimulated with LPS for 24 h before measurement of IL-8, TNF-α, IL-1β and IL-6 levels by ELISA. Each data point represents 1 patient sample. Data shown is fold change with respect to samples not treated with LPS and respective p-values shown are samples pre-treated with RSV compared to non-treated samples. Statistical analysis was performed using two-tailed paired t test.

Transrectal ultrasound (TRUS)-guided prostate biopsy is performed during a digital rectal exam on patients displaying an abnormal Prostate Specific Antigen (PSA) level or suspected prostate cancer cases. Septic complications following TRUS are well-described in about 0.1–7% of patients due to the puncture of the rectal wall with the potential for transfer of pathogens from the rectum into the sterile prostate gland or surrounding tissue^47^. We observed that monocytes from patients 24 h post-biopsy produced higher levels of pro-inflammatory cytokines including IL-8, IL-1β, IL-6, and TNFα as compared to monocytes obtained from the same patient pre-biopsy (Fig. 5A). Next, we treated monocytes isolated from post-biopsy blood samples with 2 different doses of RSV for 24 h and observed a significant dose-dependent inhibition of the production of all four cytokines (Fig. 5B). To simulate the potential prophylactic role of RSV, we used monocytes from healthy donors and pre-treated them with RSV before exposure to LPS. Indeed, there was a significant reduction in IL-8, IL-1β, IL-6 and TNFα production in monocytes exposed to 40 µM RSV (Fig. 5C). Together, these data suggest that RSV impedes the production of inflammatory cytokines following an infective stimulus (therapeutic setting), as well as when added prior to LPS exposure (prophylactic setting).

### Potential role of RSV-induced autophagy in inflammation regulation through MyD88 clearance

**Figure 6:**
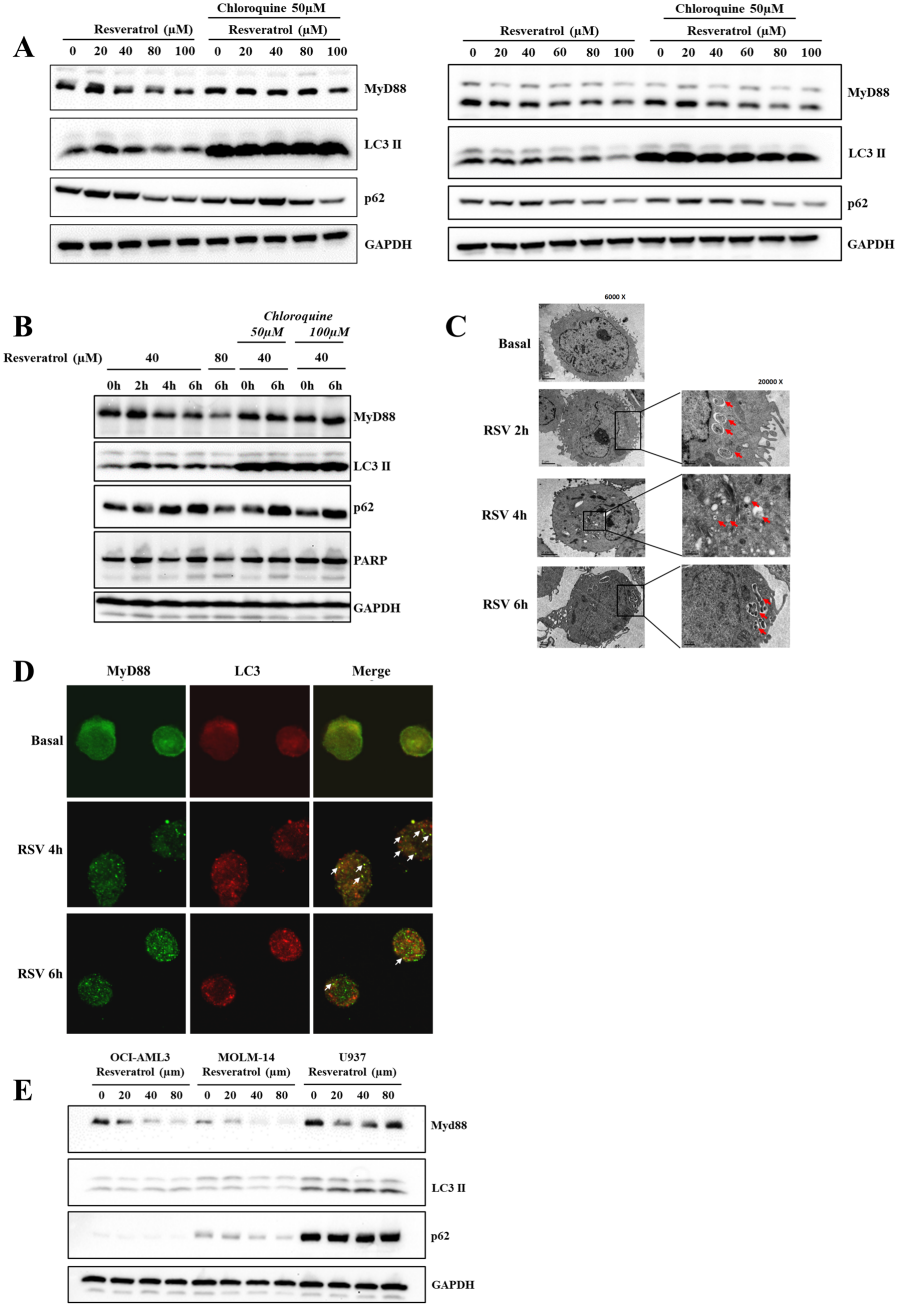
RSV treatment decreases MyD88 protein level. **(A**) U937 cells were treated with increasing concentrations of resveratrol ranging from 0 to 100 µM for 6 h with or without pre-incubation of chloroquine 50 µM for 1 h. Western blot analysis of p62, MyD88, LC3 was performed using GAPDH as loading control. (**B**) U937 cells were treated with 40 µM RSV for 0, 2, 4 and 6 h, with 80 µM RSV for 6 h, or with 40 µM RSV for 0 and 6 h plus chloroquine 50 or 100 µM. Western blot analysis of p62, MyD88, LC3 was performed using GAPDH as loading control and PARP to verify that apoptosis was not triggered by the treatment. (**C**) Electron microscopy of human monocytic U-937 cells untreated or treated with 40 µM RSV for 2 h, 6 h or 18 h. Red arrows indicate the autophagic vacuole structures. Pictures are representatives of three independent experiments. (**D**) Detection by confocal microscopy of MyD88 (green) and LC3 (red) in monocytic U-937 cells treated with or without RSV for 4 h or 6 h. White arrows indicate co-localization. Data are representative of two independent experiments. **(E**) OCI-AML3, MOLM-14 and U937 cells were treated with RSV concentrations of 0, 20, 40 and 80 µM for 6 h to assess the effect of RSV on MyD88 downregulation in different cell lines. Western blot analysis of p62, MyD88, LC3 was performed using GAPDH as loading control. Data are representative of three independent experiments. Note: western blots were performed on the same membranes cut in different strips based on molecular weight to probe for the desired targets. Data shown in C and D were derived from Dr. Wang Binbin PhD Thesis ^36^.

LPS induced pro-inflammatory signaling is a function of TLR signaling and involves the adaptor protein MyD88, which works upstream of PLD. Pertaining to the existing literature and our results, we next asked if RSV-mediated inhibition of LPS signaling was mediated through an effect on MyD88. Indeed, we observed that RSV treatment for 6 h decreased MyD88 level in U937 cells in a dose dependent manner (Fig. 6A, S2A). Furthermore, we observed that the decrease occurred only after 4 h of treatment (Fig. 6B), which suggests that this effect is temporally not upstream of the cytokine reduction induced by RSV. These data show that the diminution in the protein levels of MyD88 induced upon RSV exposure may not be the primary event leading to a reduction of cytokines production but rather a secondary late event. This nonetheless remains an interesting result as it suggests a dual regulatory effect of RSV on systemic inflammatory response. Of note, our results also showed that exposure of human monocytes to RSV alone (in the absence of LPS) induced time-dependent degradation of MyD88 (Fig. S2B, left panel) that correlated with the accumulation of the autophagic marker LC3-II and evidence of efficient autophagic flux, as indicated by the time-dependent degradation of p62 in the U937 monocytic cell line (Fig. 6A, 6B and S2B^36^, right panel). Intrigued by these findings, we next asked if the downregulation of MyD88 was a function of RSV-induced autophagic degradation. To do so, cells were pre-incubated with autophagy inhibitors chloroquine for 2 h and 3-methyladenine (3MA) for 1 h, before exposure to RSV for up to 6 h. Inhibiting autophagy apparently reduced RSV-induced degradation of MyD88 (Fig. 6A, 6B and S2C^36^). The blocking of autophagy was evidenced by the accumulation of p62 even in the presence of RSV (Fig. 6B and S2C^36^). Furthermore, to rule out the possibility that RSV-induced effect on MyD88 was a function of proteasomal degradation, U937 cells were exposed to the proteasome inhibitors, lactacystin (10 µM) or MG132 (5 µM), the lysosomal inhibitor E64D (10 µg/ml) + pepstatin A (10 µg/ml) or the autophagy inhibitors chloroquine (50 µM) and bafilomycin A1 (200 nM). While, addition of chloroquine, bafilomycin A1 or lactacystin to inhibit autophagy and lysosomal activity resulted in partial rescue of MyD88 levels, the proteasomal inhibitors had no effect on RSV-induced MyD88 degradation (Fig. S2D^36^ and S2E). This experiment was also repeated using human primary monocytes and the results showed that RSV-mediated MyD88 downregulation was alleviated in the presence of either chloroquine or E64D + pepstatin A (Fig. S2D^36^, right panel). To test autophagosome formation, U-937 monocytic cells were pre-treated with 40 µM RSV before observation using transmission electron microscopy. The amount of double-membrane vesicles characteristic of autophagosomes appeared to increase in U-937 cells treated with 40 µM RSV for 2–18 h compared to untreated cells (Fig. 6C)^36^. We also looked at the intracellular localization of MyD88 and LC3 in U-937 cells treated with RSV using confocal fluorescence microscopy. We observed an increased number of yellow puncta potentially indicating co-localization of MyD88 (green) and LC3 (red) in U-937 cells treated with RSV for 4 h, which was reduced at 6 h, potentially indicative of the active degradation of MyD88 through the lysosomes (Fig. 6D)^36^. This effect was also tested in the acute myeloid leukemia cell lines AMI-AML3 and MOLM-14 and confirmed that RSV treatment induces a significant decrease in MyD88 level (Fig. 6E). Collectively, using a variety of experimental set ups, results show that RSV treatment reproducibly results in a decrease in MyD88 levels. However, it is noteworthy that the rescue of MyD88 with inhibitors of autophagy such as chloroquine was not always very strong, thereby indicating that despite the observations associating RSV-induced autophagy to its effect on MyD88 levels, mechanisms other than autophagy might be operative in RSV-mediated downregulation of MyD88 (Fig. 6A right panel, 6B and S2E).

## Discussion

Understanding the molecular mechanisms that control LPS-mediated inflammatory responses has important implications, especially in identifying novel anti-bacterial therapeutic strategies. Despite extensive efforts to advance treatments for sepsis, numerous clinical trials have been unsuccessful. Anti-inflammatory agents such as TNFα monoclonal antibodies and nitric oxide synthase inhibitors resulted in increased lethality through potentially suppressing the host’s immune responses against the infection^48,49^. Hence, there is a need to develop new therapies in addition to antibiotics. Recently, studies have advocated that limiting the inflammation level triggered by TLRs could represent a successful strategy to fight sepsis^50^. To that end, targeting TLR4 signaling with natural antagonists have been shown to provide protection in septic models such as with Atractylenolide I treatment that reduces TNFα and IL-6 production as well as decreases ERK and NF-κB activation^51,52^. In this study, we aimed to decipher the mechanisms of the anti-inflammatory activity of RSV on the intracellular signal cascade initiated by LPS and in a murine model of sepsis.

**Figure 7:**
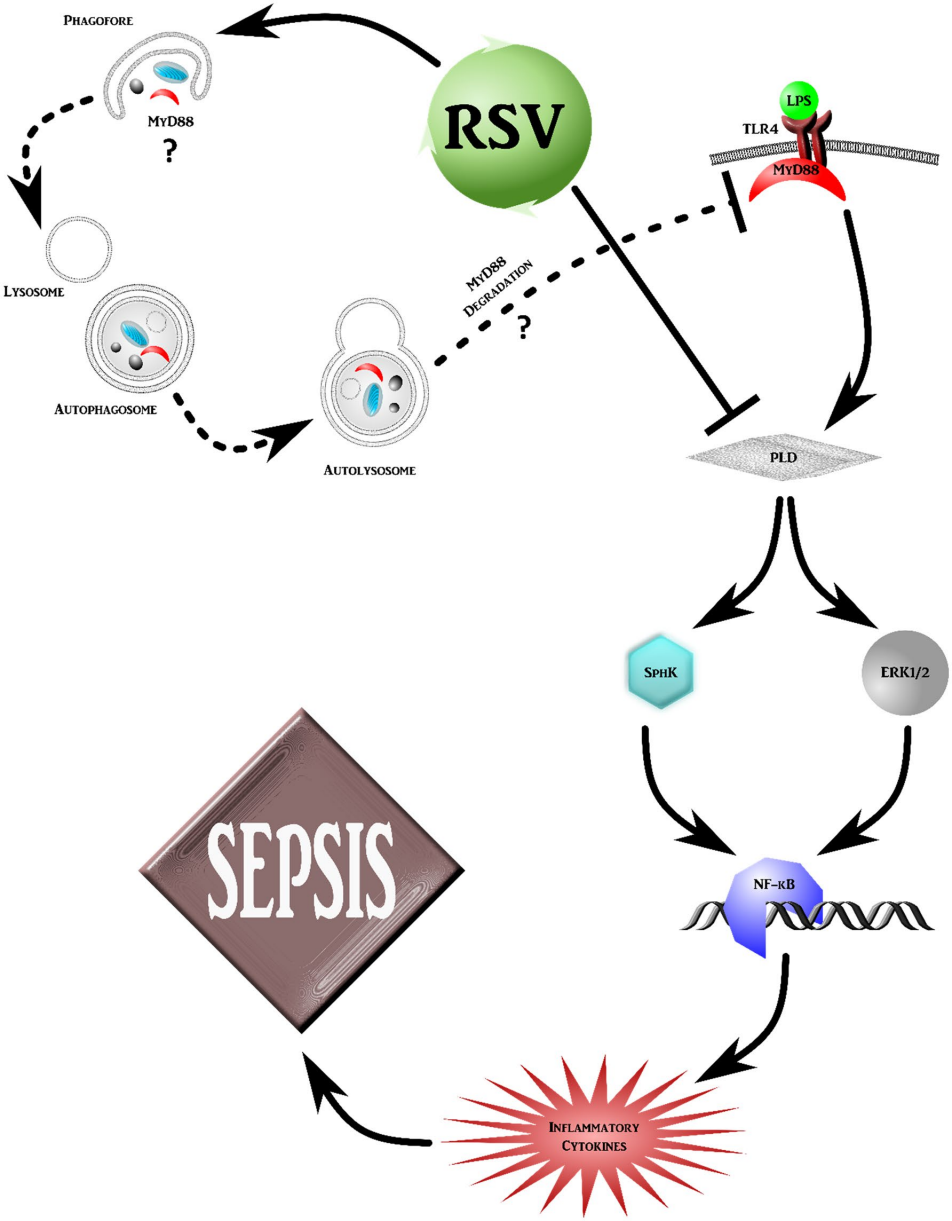
Proposed molecular mechanisms underlying RSV-mediated anti-inflammatory effects in the TLR4 signaling pathway.

Our data strongly suggest that LPS-induced SphK activation and ERK1/2 phosphorylation in monocytes is achieved via PLD activation (Fig. 3A,B). However, inhibiting ERK1/2 activity had no effect on LPS-induced SphK activation and vice versa, hence it is likely that they are independent pathways downstream of PLD that converge to activate NF-κB and control the pro-inflammatory responses in monocytes (Fig. 7).

The pathogenesis of sepsis involves in part bacterial endotoxins which triggers the production of pro-inflammatory factors by monocytes/macrophages/neutrophils sequentially thus generating early (e.g. TNFα, IL-1β) and late inflammatory responses (e.g. HMGB1)^53^. The understanding of the involvement of these pro-inflammatory mediators in the onset of sepsis have greatly improved. Nevertheless, one of the main challenge in developing strategies targeting the early phase of cytokine production is the narrow therapeutic window. Therefore, many studies have explored the possibility of modulating the level of HMGB1 as it can be passively released by apoptotic and necrotic cells, and is actively produced by phagocytes following endotoxin stimulation. In addition, it can induce the synthesis of TNFα, IL-1β, and IL-6 in monocytes/macrophages^5,54^. In this study, we showed that RSV is able to reduce HMGB1 production by monocytes in vitro as well as systemically in vivo in the CLP mice model. One potential mechanism by which RSV could inhibit HMGB1 release is by preventing HMGB1 nuclear-cytoplasmic translocation through up-regulating SIRT1 in a sepsis-induced liver injury mouse model^55^.

Neutrophils intervention is a major step in the elimination of bacterial pathogens, but it is also involved in organ failure observed in sepsis. Some investigations using animal models of sepsis indeed showed that reducing the recruitment of neutrophils to the infection site resulted in an improved survival^56,57^. Our results show that RSV can diminish neutrophil infiltration into the peritoneum in our murine model of CLP-induced sepsis. This might be due to the reduced chemokine secretion for recruiting neutrophils in the peritoneal cavity. Despite the reduced recruitment of neutrophils into the peritoneum, a concomitant bacterial clearance is observed in the RSV-treated group, both in the blood and peritoneal lavage. The lower bacteria count is likely attributed to the anti-microbial properties of RSV^58^. Furthermore, blocking gamma interferon (IFNγ) in a CLP mouse model was shown to decrease bacterial load by facilitating fibrin deposition and tissue repair, thus alleviating systemic inflammation by promoting wound healing of the punctured cecum and limiting bacterial spread^59^. Treatment with RSV in our CLP model moderately reduced IFNγ in the peritoneal cavity (Fig. S1), which suggests a plausible cause for the anti-bacterial effect observed in this model system. Taken together, these results show that RSV limits the activation of important signaling molecules (PLD, SphK1, ERK1/2 and NFκB) stimulated by LPS which leads to the mitigation of pro-inflammatory cytokines and chemokines production in human primary monocytes.

In order to evaluate the therapeutic potential of RSV in sepsis, we further questioned whether in vivo injection of RSV could offer a protective effect against septic shock in a model of CLP-induced sepsis. Indeed, RSV significantly inhibits serum cytokines, chemokines and HMGB1 release as well as immune cells infiltration to the tissues, conferring protection against lethality in septic shock. Furthermore, the ability of RSV to reduce inflammatory cytokine production in human monocytes from prostate-biopsied patients suggests a possible role of reducing systemic inflammatory responses in patients undergoing prostate biopsy by administering RSV prophylactically.

Finally, we investigated a probable mechanism for the LPS inhibitory effect of RSV implicating autophagic clearance. Our data show that RSV can also modulate TLR4 mediated pro-inflammatory responses via the downregulation of MyD88. Along similar lines, genipin and artesunate have also been shown to protect against sepsis by mitigating TLR4 activation through MyD88 downregulation^60–62^. Of note, the degradation of MyD88 induced by RSV is not observed at the initial stage of RSV-induced autophagy (2 h post LPS treatment) but can only be detected after 4 h or longer treatment. This suggests that RSV treatment appears to act at two levels (different time kinetics) in regulating or mitigating the inflammatory process. There is an early inhibition of SphK activity, mediated via the ability of RSV to regulate PLD activity, and a late (upon 4 h or more treatment) effect on MyD88. It is noteworthy that autophagy has been shown to attenuate endotoxin-induced inflammatory response in the intestinal epithelium by inhibiting NF-κB activation^63^. The importance of autophagy in the release of pro-inflammatory cytokines has also been shown in mice with a ATG16L1 gene deletion in which the absence of this autophagy protein promoted an over-production of inflammatory cytokines by macrophages^64^. However, the understanding of how the autophagy machinery regulates cytokine production requires further investigation. Although, we are unclear as to the direct crosstalk between these two activities, we argue that the degradation of MyD88 induced by RSV might also impact SphK activity at a later time point but could essentially be involved in inflammation mitigation. We clearly observed the reproducibility of RSV treatment leading to a decrease in MyD88 level in a dose- and time-dependent manner (Fig. 6A,B). Our current work presents a plausible explanation that compounds such as RSV may confer beneficial anti-inflammatory effects through a biphasic biological effect (Fig. 7). First the treatment triggers an early effect (within 1 h) that alleviates cytokine production through the inhibition of PLD and downstream NF-κB and ERK signaling, followed by a rather late effect (4 h or longer exposure) leading to a decrease in MyD88 levels. While these results provide testament for the therapeutic potential of RSV against systemic inflammatory response (such as sepsis), the direct link between RSV-induced autophagy and its potential involvement in the degradation of MyD88 is worthy of further investigations.

## Conclusion

Collectively, these data provide a proof-of-concept for the therapeutic potential of RSV in models of systemic inflammation. Our findings demonstrate the inhibitory effect of RSV on TLR4-mediated pro-inflammatory cytokine production in vitro as well as highlight using in vivo models the therapeutic potential in alleviating cytokine storm associated with systemic sepsis.

## Availability of data and material

The datasets used and/or analysed during the current study are available from the corresponding author on reasonable request.

## Supplementary information

Supplementary information
