# Supplementary material for: Resveratrol attenuates TLR-4 mediated inflammation and elicits therapeutic potential in models of sepsis
Source: Sci Rep. 2020 Nov 2;10:18837. doi: 10.1038/s41598-020-74578-9 (PMC7608666; doi:10.1038/s41598-020-74578-9)
Supplement: Supplementary file 1 — Supplementary information [file 41598_2020_74578_MOESM1_ESM.pdf]

Resveratrol attenuates TLR-4 mediated inflammation and elicits therapeutic potential in models of sepsis

Binbin Wang[1,2,*], Gregory Lucien Bellot[1,3,*], Kartini Iskandar[1], Tsung Wen Chong[4], Fera Yiqian Goh[4], June Jingyi Tai[5], Herbert Schwarz[1], Siew Cheng Wong[5,6,#], and Shazib Pervaiz[1,2,7,8,#]

[1]Department of Physiology, National University of Singapore, Singapore. [2]NUS Graduate School for Integrative Sciences and Engineering, National University of Singapore. [3]Department of Hand & Reconstructive Microsurgery, University Orthopedic, Hand & Reconstructive Microsurgery Cluster, National University Health System, Singapore. [4]Department of Urology, Singapore General Hospital (SGH). [5]Singapore Immunology Network (SIgN), Agency for Science, Technology and Research (ASTAR), Singapore. [6]Department of Biochemistry, National University of Singapore, Singapore. [7]National University Cancer Institute, National University Health System, Singapore, Singapore. [8]Faculte de Medicine, University of Paris, Paris, France.

*Contributed equally to this work

[#]Correspondence: Shazib Pervaiz, Department of Physiology, Yong Loo Lin School of Medicine, National University of Singapore, Singapore 117597. E-mail: phssp@nus.edu.sg or Siew Cheng Wong, Singapore Immunology Network (SIgN), Agency for Science, Technology and Research, Singapore 138648. E-mail: wong_siew_cheng@immunol.a-star.edu.sg.

Running title: Resveratrol mitigates inflammation in sepsis

Keywords: Sepsis, Resveratrol, signaling, autophagy, monocytes

Supplementary Figure 1

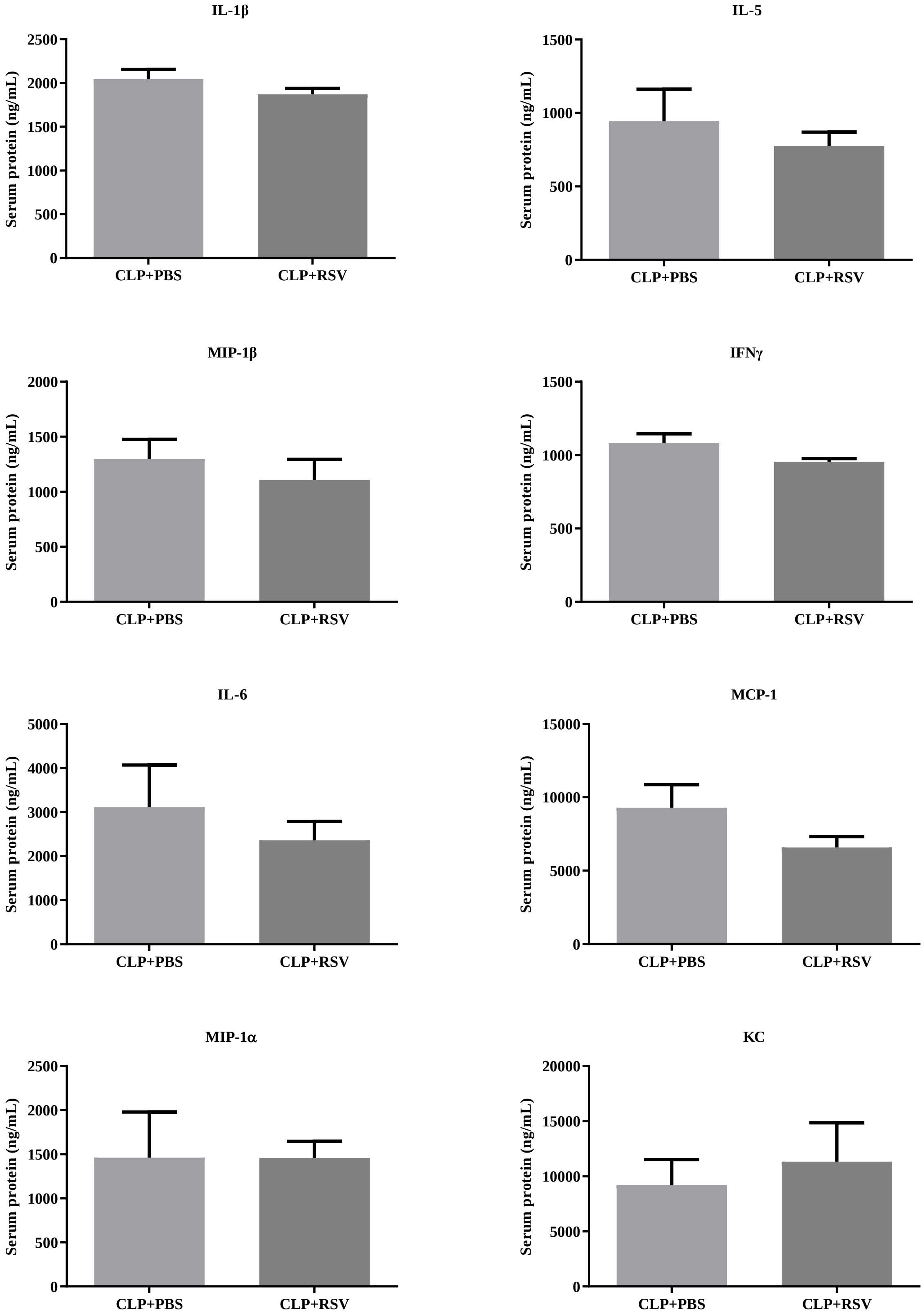

Supplementary Figure 2

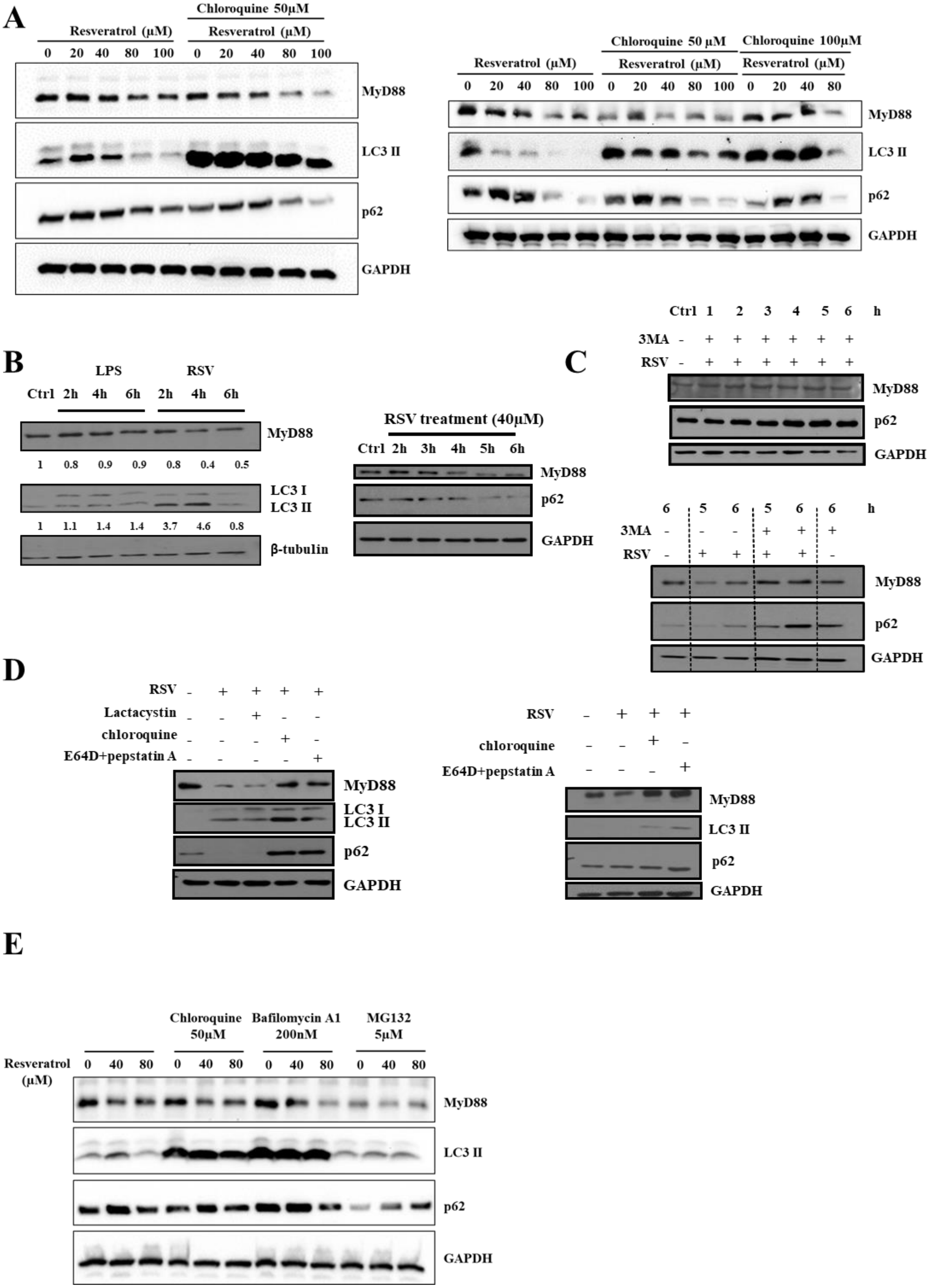

Supplementary Figures Legends

Supplementary figure 1: Levels of L-1β, IL-5, IL-6, MCP-1, IFNγ, MIP-1α, MIP-1β and CXCL1(KC) in serum of mice treated as in figure 4A, were measured 24h after CLP by ELISA. Statistical analysis did not reveal any significant variation between PBS and RSV-treated groups in these conditions. Data were derived from Dr. Wang Binbin PhD Thesis.

Supplementary figure 2: A) U937 cells were treated with increasing concentrations of resveratrol ranging from 0 to 100µM for 6h with or without pre-incubation of chloroquine 50µM or 100µM for 1h. Western blot analysis of p62, MyD88, LC3 was performed using GAPDH as loading control. **B**) Human primary monocytes were treated with 40µM RSV for the indicated time points (2-6h). Western blot analysis of MyD88, LC3 and p62 was performed using β-tubulin as loading control. **C**) Immunoblot analysis of expression of MyD88, LC3 and p62 in U-937 cells (upper panel) or human primary monocytes (lower panel) treated with 40µM RSV for 0, 2, 3, 4, 5 and 6h pre-incubated with 5mM 3MA for 1h and then treated. GAPDH was used as loading control. **D**) Immunoblot analysis of expression of p62, MyD88 and LC3 in human monocytic U-937 cells (left panel) or human primary monocytes (right panel) treated with 40µM RSV for 6h with pre-incubation of either 50µM chloroquine, 10µg/ml E64D+10µg/ml pepstatin A or 10µM lactacystin. GAPDH was used as loading control. **E**) U937 cells were treated with 0, 40 or 80µM of resveratrol for 6h alone or in presence of 80µM chloroquine, 200nM bafilomycin A1 or 5µM MG132. Western blot analysis of p62, MyD88, LC3 was performed using GAPDH as loading control. Data shown in B, C and D were derived from Dr. Wang Binbin PhD Thesis.
